# Alpha-internexin as a brain-specific neurodegeneration marker: development and validation of a novel CSF assay

**DOI:** 10.1007/s00415-025-13428-y

**Published:** 2025-10-06

**Authors:** Francisco J. Meda, Anna Dittrich, Ingmar Skoog, Silke Kern, Claire Leckey, Edward J. Wild, Ross W. Paterson, Gunnar Brinkmalm, Ulf Andreasson, Kaj Blennow, Henrik Zetterberg, Hlin Kvartsberg

**Affiliations:** 1 Department of Psychiatry and Neurochemistry, Institute of Neuroscience and Physiology, The Sahlgrenska Academy at the University of Gothenburg, Mölndal, Sweden; 2 Sahlgrenska Academy, University of Gothenburg, Neuropsychiatric Epidemiology Unit, Gothenburg, Sweden; 3 Sahlgrenska University Hospital, Department of Neuropsychiatry, Mölndal, Sweden; 4 UK Dementia Research Institute at University College London, London, UK; 5 UCL Huntington’s Disease Centre, Institute of Neurology, University College London, London, UK; 6 Clinical Neurochemistry Laboratory, Sahlgrenska University Hospital, Mölndal, Sweden; 7 Hong Kong Center for Neurodegenerative Diseases, HKCeND, Hong Kong, China; 8 Wisconsin Alzheimer’s Disease Research Center, School of Medicine and Public Health, University of Wisconsin, Madison, WI USA; 9 Department of Pathology and Laboratory Medicine, School of Medicine and Public Health, University of Wisconsin, Madison, WI USA; 10 Department of Neurodegenerative Disease, Dementia Research Centre, UCL Institute of Neurology, Queen Square, London, UK; 11 Centre for Brain Research, Indian Institute of Science, Bangalore, India

**Keywords:** Alpha-internexin, Neurofilaments, Immunoassay, Cerebrospinal fluid

## Abstract

**Background:**

Alpha-internexin (AINX) is a type IV intermediate filament alongside the neurofilament triplet proteins and periferin. Despite its homologies, the key difference is that AINX is central nervous system-specific. The purpose of this study was to develop an immunoassay for cerebrospinal fluid (CSF) AINX, quantify it in patients with neurodegenerative diseases, and compare its diagnostic performance with neurofilament light (NfL).

**Methods:**

Monoclonal antibodies were generated and characterized by immunoprecipitation followed by mass spectrometry and ELISA, for specificity to the target analyte and cross-reactivity. Single molecule array (Simoa) technology was the selected platform for assay development. The assay was validated according to pre-established parameters and AINX performance as a biomarker was tested in two independent cohorts (Gothenburg and University College London).

**Results:**

Two highly specific antibodies were generated (B15 and Ina1) and used to develop a robust assay in all analytical validation parameters tested. The quantitative range of the assay was 0.137–140 pg/mL. In the Gothenburg cohort, AINX and NfL showed similar diagnostic performance, but the correlation between biomarkers was diagnosis dependent. In the University College London cohort, AINX and NfL showed similar trends across the different neurodegenerative diseases, with strong correlation between markers.

**Conclusions:**

We developed a highly sensitive and specific immunoassay for AINX in CSF. AINX showed similar diagnostic performance and high correlation with CSF NfL. Further research should focus on describing its role in specific disorders, as well as evaluate its potential as a blood-based biomarker.

**Supplementary Information:**

The online version contains supplementary material available at 10.1007/s00415-025-13428-y.

## Introduction

Alpha-internexin (AINX) is a 66 kDa protein, similar in structure to the neurofilament triplet proteins (NFTPs), neurofilament light (NfL), neurofilament medium (NfM), and neurofilament heavy (NfH) with a conserved mid coiled-coil rod domain, flanked by an N-terminal head and C-terminus tail domain making it a part of the type IV intermediate filament family [1, 2]. It is mainly expressed in the developing brain where it is a key factor in intermediate filament formation [3]. With its ability to homo-oligomerize, similarly to neurofilament light (NfL), AINX creates the backbone for intermediate filament formation [4, 5]. Studies suggest that AINX exists in similar abundance as the NFTPs and with relatively fixed stoichiometry of 2:1 (NfL:AINX) in rat optic nerve with similar turnover rates [6], and in mouse olfactory bulb at the embryonic and post-natal stages [7].

AINX is present across the brain, with especially high expression in cerebellar granule cells processes [8]. Recent case studies reported distinct AINX immunoreactivity throughout the brain, including more pronounced reactivity in entorhinal cortex, amygdala, superior temporal gyrus, globus pallidus, and locus coeruleus [9, 10]. Outside the brain, it is only reported to be expressed at RNA and protein level in endocrine tissues more specifically the pituitary and adrenal glands [11], and different types of neuroendocrine tumors, with lower levels of AINX being associated with advanced stage of pancreatic neuroendocrine tumors [12].

Despite being involved in the building of the neuronal filament network in the developing brain, knockout of AINX in mice does not lead to abnormal phenotypes, with NfL taking over most of its functions, thus not interfering with the normal brain development [13]. In disease, AINX is present in the inclusion bodies of neuronal intermediate filament inclusion disease, together with the NFTPs [14, 15].

Out of all the NFTPs, NfL is the most established biomarker for general neurodegeneration providing an indirect measure of axonal damage in biofluids, such as blood and cerebrospinal fluid (CSF) [16]. Several studies have described and quantified NfL either in CSF or plasma using different assays on different platforms (ELISA, Simoa, mass spectrometry) [16–18]. Despite its similarities to NfL, AINX has one major difference from the other intermediate filament proteins; it is central nervous system (CNS)-specific [6]. Previous studies show by SDS-PAGE and immunoblotting that, in mice, AINX is present in the corpus callosum, optic nerve, and spinal cord, but not in the sciatic nerve [19]. This opens the possibility for a biomarker specific for CNS neurodegeneration.

With this study, we intended to fill an important gap in the understanding of AINX in biofluids, by developing monoclonal antibodies and generating an immunoassay using the Simoa platform for its detection in CSF. We undertook an initial examination of the assay’s performance in healthy controls and patients with different neurodegenerative diseases and compared our findings to NfL levels in CSF.

## Methods

### Antibody development

Antibodies were generated by the immunization of mice with recombinant AINX peptides targeting the coil domain of the protein (aa 100–150 and aa 186–208). These peptides target the regions where there is the least sequence homology with the NFTPs. After that, titers were tested, the spleen was removed, and B cells fused with the myeloma cell line SP2/0 following standard procedures. After screening the cell media for AINX antibodies, the clones that reacted to the immunizing peptides were grown, subcloned, and stored in liquid nitrogen. Antibody purification was performed in a Hitrap protein G column (GE Healthcare) according to the manufacturer’s instructions. The generated antibodies are referred to as Ina1 (aa 100-150) and B15 (aa 186-208). B15 was initially generated as an IgM, but due to the unpredictable binding characteristics of a pentameric structure antibody, prone to agglomeration that might influence the final protein measurement [20], it was sequenced, the heavy chained was changed to IgG1, and it was expressed recombinantly as an IgG.

### Antibody specificity by enzyme-linked immunosorbent assay

Direct ELISA setup was used to assess antibody cross-reactivity. Scaling molar concentrations (0.01563–1.526e-5 pmol) of AINX89-499 (in-house AINX recombinant protein, 0.01–7.2 ng/mL) and in-house NfL (0.01–9.77 ng/mL), NfM (0.02–16.1 ng/mL), and NfH (0.02–17.5 ng/mL) extracted from bovine tissue in bicarbonate buffer (50 mM NaHCO_3_, pH 9.6) were directly coated on 96-well microtiter plates and incubated over-night at 4 °C (100 μL/well). Both antibodies were applied at a concentration of 0.5 µg/mL and secondary anti-mouse IgG-horse radish peroxidase (HRP) linked antibody was used to identify protein-antibody binding. Following a 2.5 h incubation period at room temperature (RT), 3,3’,5,5’-Tetramethylbenzidine (TMB ONE, Kem En Tech Nordic; 100 μL/well) was added to act as substrate and generate a colorimetric signal. Finally, after 30 min incubating in the dark, the reaction was stopped with 0.2 M H_2_SO_4_ (100 μL/well), and the absorbance measured at 450 nm (with 650 nm reference) using an ELISA plate reader (Sunrise, Tecan Trading AG). For sandwich ELISA, a similar protocol was used, changing the plate coating to 1 μg/mL B15 IgG. After over-night incubation, the analytes (NfL, NfH and AINX) were added in serial dilutions (0.305–5000 pg/mL) and incubated for 3 h followed by addition of 1 μg/mL Ina1 detector. After the addition of secondary anti-mouse IgG-HRP, all steps were equal as previously described.

### Immunoprecipitation

The antibodies were characterized by immunoprecipitation (IP) of sodium dodecyl sulfate (SDS) soluble brain extracts (Supplementary data 1, extraction protocol adapted from previous work [21]) as well as recombinant protein (AINX89–499, in-house, adapted from previous protocol [22]), followed by mass spectrometry detection. Ten milligrams of the SDS soluble fraction of human brain protein, previously extracted, and 2 pmol of recombinant AINX89-499 protein were thawed and used to perform AINX extraction by IP. Both antibodies were coated on beads (Dynabeads M-280 sheep anti-mouse IgG, Thermo Fisher Scientific) using 8 µg/50 µL of beads and incubated for 1 h at RT followed by a 1 h incubation with Rotiblock (Carl Roth) and 3 washes with phosphate-buffered saline (PBS). Samples were diluted to the appropriate concentration, and incubated together with 50 µL of antibody-bound beads in 0.2% (v/v) Triton X-100 for 1 h. Sample volume was reduced to 1 ml and transferred to a KingFisher magnetic particle processor (Thermo Fisher Scientific). The process consists of serial washing of the protein bound to beads with PBS 0.2% TritonX-100, PBS, ammonium bicarbonate (AMBIC, 50 mM), and elution of the bound protein in 0.5% (v/v) formic acid (FA). After the samples were speed-vac-dried and resuspended in AMBIC, tryptic digestion was performed by adding 35 ng of trypsin (sequencing grade, Promega) and incubated over-night at 37 °C. After 16 h, proteolysis was quenched with 0.1% (v/v) trifluoroacetic acid, samples speed-vac-dried, and stored at −20 °C until further analysis.

### Liquid chromatography–tandem mass spectrometry

Nanoflow liquid chromatography coupled to electrospray ionization hybrid quadrupole-orbitrap tandem mass spectrometry (Dionex Ultimate 3000 system and Q Exactive, both Thermo Fisher Scientific) was performed in a similar way as described previously [23]. Samples reconstituted in 7 µL 8% FA/8% acetonitrile in water (v/v/v) were loaded onto an Acclaim PepMap 100 C18 trap column (length: 20 mm; inner diameter: 7 μm; particle size: 3 μm; pore size: 100 Å) for online desalting, and subsequently onto a reversed-phase Acclaim PepMap RSLC column (length: 150 mm, inner diameter: 75 μm; particle size: 2 μm; pore size: 100 Å) for separation (both Thermo Fisher Scientific). Mobile phases were 0.1% FA in water (v/v) (A) and 0.1% FA/84% acetonitrile in water (v/v/v) (B). Separation was performed at a flow rate of 300 nL/min using a linear gradient of 3–40% B for 50 min at 30 °C. The mass spectrometer was operated in positive ion mode and set to acquire spectra between m/z 350 and 1800. Mass spectrometry and tandem mass spectrometry acquisitions were both obtained at a resolution setting of 70,000 using 1 microscan. Tandem mass spectrometry acquisitions were obtained using higher-energy collisional dissociation fragmentation using a normalized collision energy setting of 25, target values of 106, and maximum injection time of 250 ms. Database search was performed with Mascot Deamon (v2.6.0) using Mascot Distiller (v2.6.3.0) for isotope and charge deconvolution and Mascot Database (v2.6.1) to search against the Uniprot database. All fragment mass spectra were also evaluated manually.

### AINX immunoassay development

The immunoassay was developed on the ultra-sensitive Simoa HD-X Analyzer (Quanterix). B15 antibody was bound to dyed magnetic beads (Quanterix), used as the capture antibody and Ina1 was biotinylated (Thermo Fisher) and used as detector antibody. In-house AINX89-499 recombinant protein was used as calibrator for the assay. Calibrator, detector, and samples were diluted in homebrew diluent (Quanterix) with added 10 ug/mL of TRUBlock (Meridian Bioscience).

All samples used for validation procedures were obtained from the Clinical Neurochemistry Laboratory, Sahlgrenska University Hospital, Mölndal, Sweden, pooled, aliquoted, and kept at −80 °C. Before analysis, samples were thawed at RT and vortexed at 2000 rpm for 30 s. Validation procedures were performed as per previous published research [24] and clinical measurements obtained in a blinded and randomized way at the University of Gothenburg.

### Validation procedures

De-identified CSF samples from the clinical routine at the Clinical Neurochemistry Laboratory, Sahlgrenska University Hospital were used to make pools for all optimization and validation steps except for sample stability, where four fresh de-identified CSF samples were used for each validation parameter.

### Repeatability and intermediate precision

CSF from de-identified patients was screened for AINX and pooled into two batches with distinct concentrations. The first pool (~27 pg/mL) was used as high concentration quality control (QC) and the second pool (~2 pg/mL) was used as low concentration QC. Both samples were run in duplicates five times on five different days.

### Parallelism/spike recovery

For parallelism testing, four de-identified samples with concentration between 5.56 and 100 pg/mL were serial diluted up to 32-fold in analysis. For spike recovery, 5 samples were aliquoted 4 times and spiked with 3 different concentrations of recombinant protein calibrator (final theoretical concentration of analyte: 1.75, 3, or 7 pg/mL) in a ratio of 1-part spike, 6-part sample before appropriate dilution. To the last aliquot, the same volume of sample diluent was added. Both validation parameters were accepted when the deviation from the mean was less than 15%.

### Measurement range

The measuring range was defined, considering data from five different calibration curves from five runs performed at unique time points, using 4-parameter logistic regression with 1/Y^2^ weighting. This definition was based on calculated concentrations of AINX in CSF, and when the relative deviation from the expected concentration in the calibration curve was below 15%. Lower limit of quantification (LLOQ) was determined by testing *n* = 16 blanks (sample diluent) and calculated based on the result of 10 standard deviations above the mean signal of the blanks.

### Sample stability

Three types of conditions for sample stability were tested: freeze/thaw cycles, RT, and 4 °C. All samples were collected fresh from clinical routine at the Clinical Neurochemistry Laboratory, Sahlgrenska University Hospital and aliquoted for different purposes. In short, for freeze/thaw cycling, four samples were aliquoted six times where five aliquots underwent 1–5 cycles with one kept at −80 °C to use as reference. For each cycle, samples were thawed for 1.5 h, vortexed, lid opened, and put back at −80 °C until the next freeze/thaw cycle. When the required number of cycles was achieved, the samples were kept at −80 °C until further analysis.

For RT and 4 °C stability, the procedure was the same, CSF was aliquoted six times, and samples were left on a bench exposed to daylight or in the fridge up to 1 week. After each corresponding exposure period, the samples were stored at −80 °C until further analysis.

### Blood interference

To evaluate potential interference of blood leaking into CSF, four de-identified CSF samples were divided into four aliquots. Fresh blood was collected and diluted in NaCl (154 mM) before spiking (1-part blood, 19-part CSF) three aliquots from each individual in different volume/volume concentrations (0.1; 0.02; 0.004%). The remaining aliquots had the same volume of NaCl added and acted as the reference. After spiking or addition of NaCl buffer, samples were vortexed and centrifuged 10 min at 4000 g, and supernatants were collected and stored at −80 °C until analysis.

### Study participants

In this study, two cohorts were included. The Gothenburg cohort included Alzheimer’s Disease (AD) patient samples (*n* = 20) obtained from the H70 Clinical Studies at Sahlgrenska University Hospital, and cognitively healthy controls (*n* = 20) from the Gothenburg H70 Birth Cohort Study at the University of Gothenburg (Table 2) [25, 26].

The University College London, Institute of Neurology (UCL ION) cohort consisted in healthy controls (*n* = 5), AD (*n* = 15), corticobasal syndrome (CBS) (*n* = 4), Huntington’s disease (HD) (*n* = 9), semantic dementia (SD) (*n* = 5), dementia with Lewy bodies (DLB) (*n* = 18), multiple sclerosis (MS) (*n* = 9), and behavioral variant frontotemporal dementia (bvFTD) (*n* = 2) with patient recruitment, sample collection, and patient diagnosis as previously described [27].

### Statistical analysis

Analysis of covariance (ANCOVA) followed by post hoc Bonferroni test was used to evaluate comparisons between groups in both cohorts. Spearman correlations between NfL and AINX levels were used in both datasets. *P* values lower than 0.05 were considered significant. In the UCL ION cohort, bvFTD was excluded from all statistical analysis due to limited sample size (*n* = 2). ANCOVA was performed using IBM SPSS Statistics software (v.29.0.1.1), with all plots and Spearman correlations made using GraphPad Prism (v10.4.1).

## Results

### Developed antibodies are specific for the target analyte

**Fig. 1:**
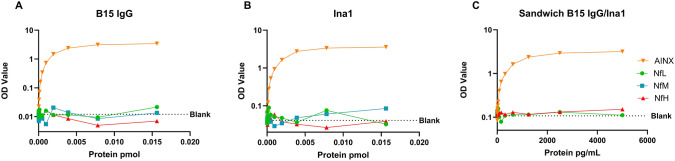
Reaction of alpha-internexin antibodies, **A)** B15 IgG, **B)** Ina1, and **C)** sandwich setup with B15 IgG as capture and Ina1 as detector, to different concentrations of neurofilament proteins. Dotted lines represent mean OD value of the blanks. Orange inverted triangles represent reaction to alpha-internexin recombinant protein (AINX89-499), green circles represent reaction to bovine neurofilament light, blue squares represent reaction to bovine neurofilament medium, and red triangles represents reaction to bovine neurofilament heavy. OD, optical density

After immunoprecipitation of SDS brain homogenates with both antibodies, liquid chromatography–mass spectrometry showed good coverage of tryptic peptides from the target analyte AINX (B15—41% and Ina1—85%). Besides this, B15 showed very little cross-reactivity with other neurofilament species, while Ina1 was less specific, showing some neurofilament homologous peptides (Supplementary data 2). However, cross-reactivity testing performed using ELISA against recombinant AINX and bovine-purified NFTPs verified that both antibodies react to AINX, but not with any NFTP, with absorbance values neighboring the blank measurement independently of concentration (Fig. 1A-B). Sandwich ELISA setup with the most specific antibody as capture (B15 IgG) and Ina1 as detector yielded similar results, proving the analyte specificity of the antibodies in this setup. (Fig. 1C).

### Method validation parameters show a sensitive and robust assay for CSF AINX

**Fig. 2:**
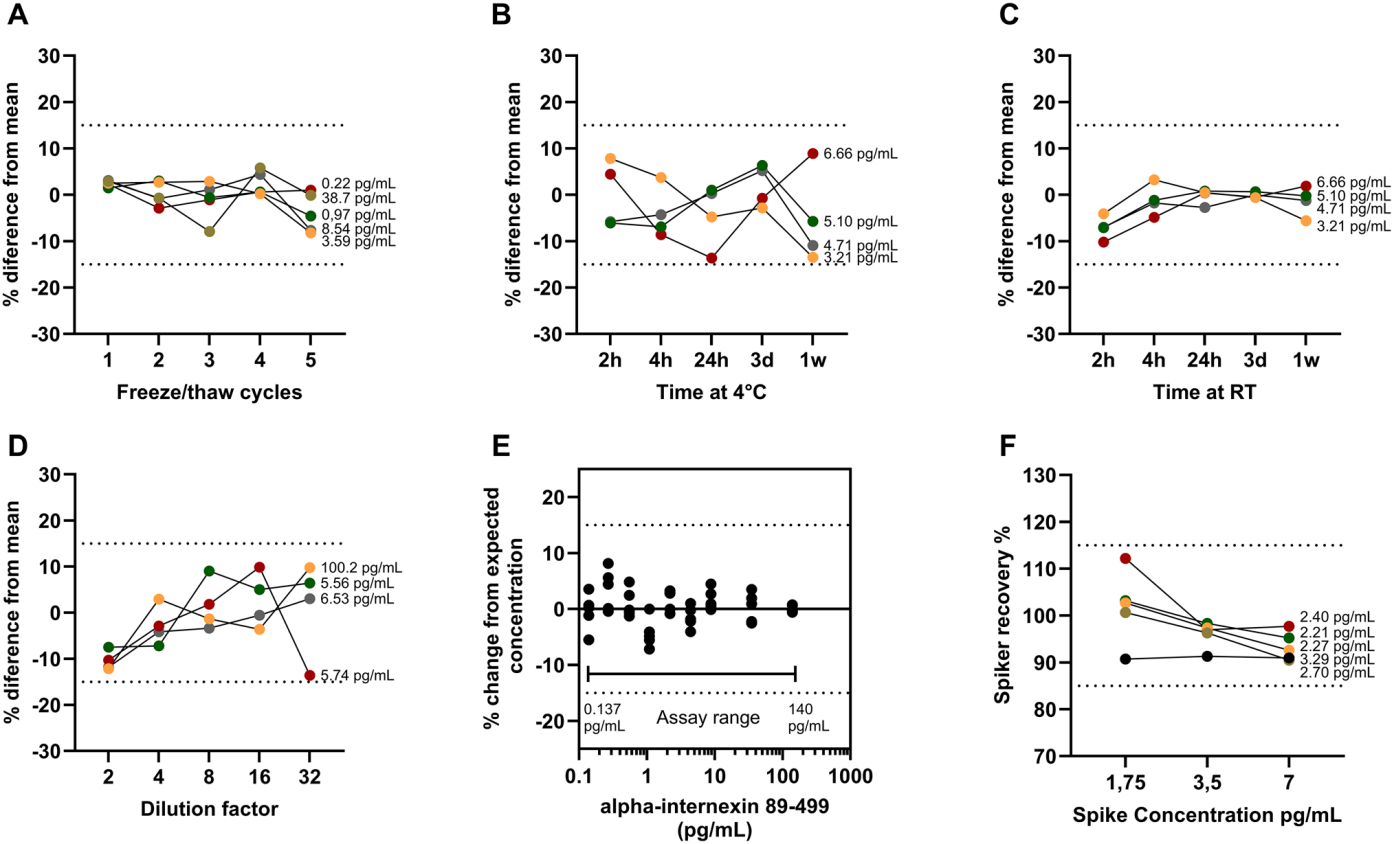
Validation parameters. **A)** Freeze/thaw cycle endurance, stability at **B)** 4 °C and **C)** room temperature. This data is plotted as percentage difference from mean. **D)** Parallelism, calculated concentrations of samples diluted until 32-fold, **E)** percentage change from expected concentration in 5 different calibration curves with defined range between 0.137 and 140 pg/mL, and **F)** spike recovery between 102 and 93.4%. Dotted lines indicate 15% uncertainty limit. Concentrations annotated in the plots represent initial concentrations of the samples tested

Repeatability for samples with different concentrations stayed below 3.4% for samples with mean concentration of 27.6 pg/mL (high control) and 2.7% for samples with mean concentration of 2.01 pg/mL (low control). Intermediate precision stayed below 4.5% for the high control and 10.4% for the low control (Table 1). CSF AINX was stable at room temperature and 4 °C up to a week and withstood up to five freeze thaw cycles (Fig. 2A-C). Parallelism exhibited stability in calculated concentrations up to 32-fold dilution (Fig. 2D). Based on average sample concentrations, the selected dilution for the assay was set at two-fold. Measurement range was established as a 10-point calibration curve ranging from 0.137 to 140 pg/mL. Deviations from expected concentrations were calculated for all points and stayed between −7.2% and 8.1% (Fig. 2E). Spike recovery with three different spike concentrations (1.75, 3.5, 7 pg/mL) showed good recovery averaging on 102, 96.1, and 93.4%, respectively to the spike concentration (Fig. 2F). Calculated from *n* = 16 blank measurements, the LLOQ of this assay was determined to be 0.113 pg/mL (data not shown). The range of the assay was established to be between the highest point in the calibration curve (ULOQ = 140 pg/mL) and the lowest that was still above LLOQ (0.137 pg/mL). Furthermore, blood contamination into CSF did not interfere with AINX concentrations, with fluctuations between −12.0 and 7.20% (Supplementary data 3).

**Table 1:** Repeatability and intermediate precision of CSF alpha-internexin detected by the developed Simoa assay

			Repeatability		Intermediate precision	
Sample dilution 1:2	Mean concentration (pg/mL)	N	CVr (%)	SDr	CVrw (%)	SDrw
High control CSF	27.6	5	3.4	0.95	4.5	1.3
Low control CSF	2.01	5	2.7	0.05	10.4	0.21

CSF, cerebrospinal fluid; CV, coefficient of variation; SD, standard deviation; r, repeatability; rw, intermediate precision

### Alpha-internexin shows distinct characteristics compared to neurofilament light

**Fig. 3:**
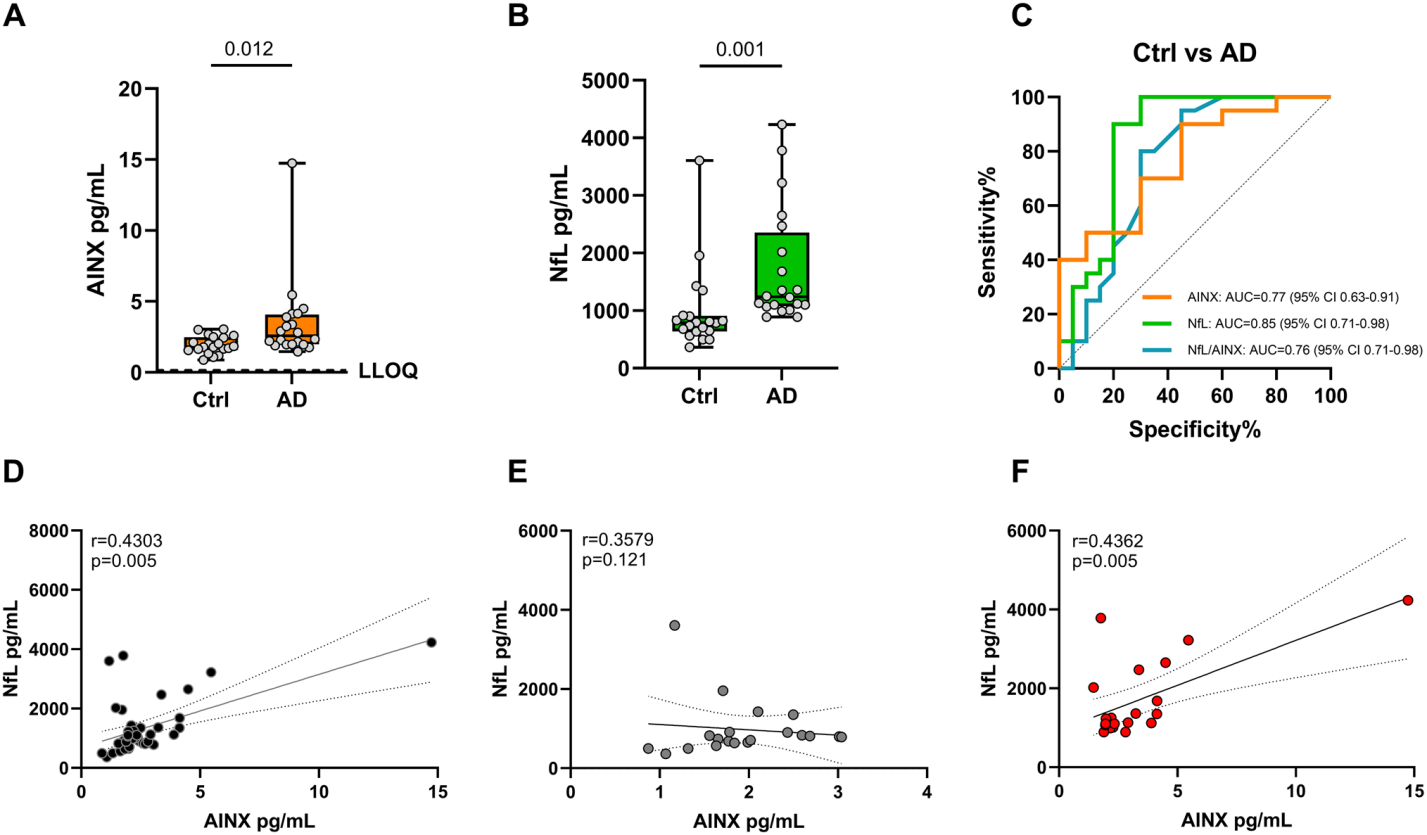
Alpha-internexin and neurofilament light concentrations (pg/mL) in the Gothenburg cohort. Boxplots represent median and IQR for **A)** alpha-internexin and **B)** neurofilament light by diagnosis group. Dashed line represents AINX assay LLOQ. **C)** Receiver-operating characteristics curves for AINX, NfL and NfL/AINX to discriminate between Ctrl and AD. Spearman correlations between alpha-internexin and neurofilament light in **D)** the whole cohort, **E)** the control group, and **F)** the Alzheimer’s group. Group comparisons made using age- and sex-adjusted analysis of covariance with Bonferroni post hoc test. Full lines represent fitted linear regression and dotted lines represent 95% confidence intervals. IQR, interquartile range; AUC, area under the curve; Ctrl, controls (*n* = 20); AD, Alzheimer’s disease (*n* = 20); AINX, alpha-internexin; NfL, neurofilament light; LLOQ, lower limit of quantification; r, Spearman correlation coefficient

The Gothenburg cohort was used as a discovery cohort where the performance of AINX and NfL as biomarkers was compared in 20 controls (Ctrl) and 20 AD samples. AINX significantly separated Ctrl from AD (*p* = 0.012) (Fig. 3A). Similarly, NfL also significantly distinguishes Ctrl from AD (*p* = 0.001) (Fig. 3B) with relative comparisons between the biomarkers showing the same trend. The absolute concentrations of AINX were much lower compared to NfL, within the single digit pg/mL (Table 2). Receiver-operating characteristics (ROC) analysis was used to evaluate case–control discrimination. NfL had better accuracy to differentiate AD from Ctrl AUC = 0.85 (95% CI 0.71–0.98) than AINX AUC = 0.77 (95% CI 0.63–0.91), while NfL/AINX ratio did not improve the diagnostic performance AUC = 0.76 (95% CI 0.71–0.98) (Fig. 3C).﻿

**Table 2:** Demographics of both cohorts, Simoa AINX and NfL levels in pg/mL

Gothenburg cohort	Age at LP, mean (SD)	male %	Simoa AINX pg/mL median (IQR)	CSF NfL pg/mL median (IQR)	Simoa AINX pg/mL mean (95% CI)	CSF NfL pg/mL mean (95% CI)
Healthy controls (*n* = 20)	70^a^	58	**1.811** (1.577–2.483)	**791** (640—912)	**1.784**^c^ (0.818–2.749)	**873**^c^ (489–1247)
Alzheimer's disease (*n* = 20)	59.6 (9.24)	67	**2.570** (1.968–4.069)	**1240** (1078—2358)	**3.613**^c^ (2.648–4.578)	**1832**^c^ (1447–2216)
UCL ION cohort				Simoa CSF NfL pg/mL median (IQR)		Simoa CSF NfL pg/mL mean (95% CI)
Healthy controls (*n* = 5)	64.7 (6.26)	53	**0.7027** (0.4187—1.962)	**636.9** (438.9—991.3)	**1.134**^d^ (−0.617–2.885)	**665.4**^d^ (−11.30–1342)
Alzheimer's disease (*n* = 15)	59.6 (9.24)	60	**3.225** (1.846—5.282)	**1007** (700—1317)	**3.633**^d^ (2.580–4.685)	**1135**^d^ (727.7–1541)
Huntington's disease (*n* = 9)	53.4 (9.07)	66	**5.463** (3.187—7.297)	**2632** (2086—3243)	**5.537**^d^ (4.112–6.963)	**2552**^d^ (2001–3103)
Corticobasal syndrome (*n* = 4)	61.3 (8.97)	75	**3.143** (2.916—7.746)	**1368** (1072—2698)	**4.614**^d^ (1.381–7.213)	**1707**^a^ (952.9–2462)
Semantic dementia (*n* = 5)	62.0 (8.40)	100	**4.010** (2.603—5.471)	**1213** (937.8—1873)	**4.076**^d^ (2.298–5.854)	**1371**^d^ (684.2–2058)
Dementia with Lewy bodies (*n* = 18)	66.0 (8.26)	72	**1.738** (1.096—3.796)	**860.5** (696.8—1084)	**2.241**^d^ (1.266–3.216)	**969.5**^d^ (592.7–2462
Multiple sclerosis (*n* = 9)	55.8 (7.39)	55	**0.7871** (0.4253—1.023)	**616.1** (511.8—762.0)	**0.870**^d^ (−0.531–2.271)	**659.2**^d^ (117.7–1201)
bvFTD (*n* = 2)	50.0 (7.80)	100	**4.167**^b^	**3240**^b^	^b^	^b^

Bold values represent each group median and mean biomarker concentrations, respectively. LP, Lumbar puncture; SD, Standard deviation; AINX, Alpha-internexin; IQR, Interquartile range; CSF, Cerebrospinal fluid; NfL, Neurofilament light; bvFTD, Behavioral variant Frontotemporal Dementia; CI, confidence intervals

^a^All patients were 70 years old

^b^Excluded from analysis

^c^After analysis of covariance, with covariates at the following values: age = 61.45, sex = 0.6818

^d^After analysis of covariance, with covariates at the following values: age = 68.43, sex = 0.6250

However, when looking at correlations, despite the correlation coefficient being similar in all comparisons (AINX/NfL *r* = 0.430, NfL/AINX Ctrl, *r* = 0.357 and NfL/AINX AD, *r* = 0.436), we can see a spread in values responsible for lower correlation values (Fig. 3D). When we separate the samples between Ctrl and AD, the correlations are lower and non-significant in Ctrl (Fig. 3E-F).

### Alpha-internexin performs similarly to NfL across neurodegenerative disease

To further investigate the importance of AINX across neurodegenerative diseases, the UCL ION cohort was used to compare AINX levels in the neurodegenerative disease spectrum.

**Fig. 4:**
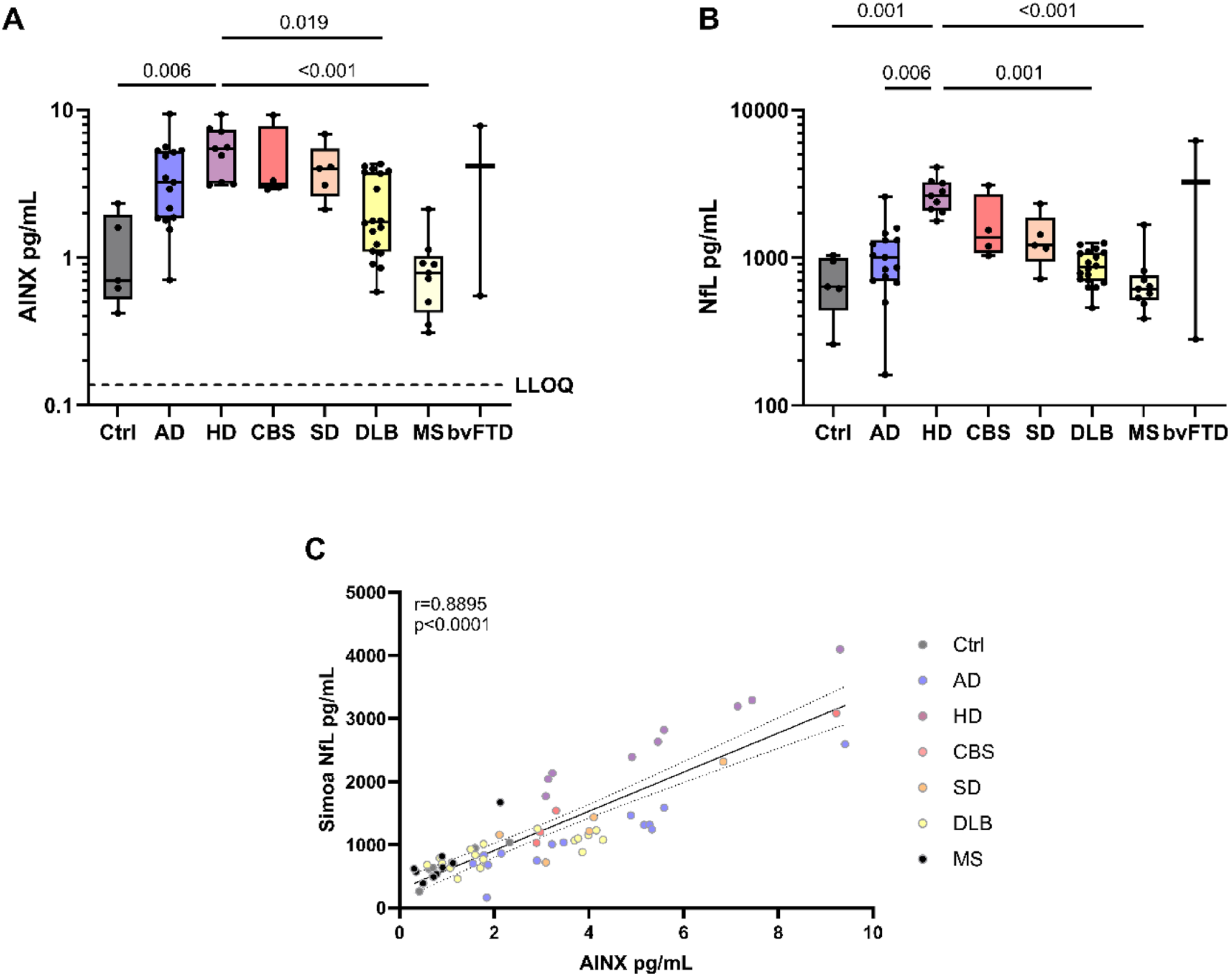
Alpha-internexin and neurofilament light concentrations (pg/mL) in the UCL ION cohort. Boxplots represent median and IQR of **A)** AINX and **B)** NfL concentrations across diagnosis groups. Dashed line represents AINX assay LLOQ. **C)** Spearman correlation between AINX and NfL in the whole data set. P values from the group comparisons made using age- and sex-adjusted analysis of covariance with Bonferroni post hoc test are shown above the lines. Full line represents fitted linear regression and dotted lines represent 95% confidence interval. IQR, interquartile range; Ctrl, controls; AD, Alzheimer’s Disease (*n* = 15); HD, Huntington’s Disease (*n* = 9); CBS, corticobasal syndrome (*n* = 4); SD, semantic dementia (*n* = 5); DLB, dementia with Lewy bodies (*n* = 18); MS, multiple sclerosis (*n* = 9); bvFTD: behavioral variant frontotemporal dementia (*n* = 2); AINX, alpha-internexin; NfL, neurofilament light; LLOQ, lower limit of quantification

The highest NfL levels were seen in HD patients, where they were significantly higher than Ctrl, MS, DLB, and AD (HD vs Ctrl, *p* = 0.001; vs MS, *p* < 0.001; vs DLB, *p* = 0.001; vs AD, *p* = 0.006) (Fig. 4A). AINX levels showed a similar pattern: significantly higher in HD compared to Ctrl and MS (HD vs Ctrl, *p* = 0.006; vs MS *p* < 0.001). Additionally, AINX was higher in MS than AD patients (*p* = 0.019) (Table 2, Fig. 4B).

Comparing the distribution of both biomarkers between groups, we found that the disease distribution of the samples across groups followed the same trend. Spearman rank correlations were used to determine the relationship between NfL and AINX in the whole cohort as well as between individual diagnosis groups. Correlations as a whole showed little variance and strong positive correlation between the two biomarkers, independently of disease diagnosis (*r* = 0.8895, *p* < 0.0001) (Fig. 4C). However, splitting the correlations based on disease diagnosis showed that, while most groups had high correlation coefficients (Ctrl, r = 1.00; AD, r = 0.910; HD, r = 1.00; CBS, r = 1.00; SD, r = 0.900), pushing the whole correlation coefficient up, DLB and MS have slightly lower coefficients (DLB, r = 0.762; MS, r = 0.683) (Supplementary data 4). Diving into DLB specifically, in this cohort, correlations between these two biomarkers clearly generate two distinct groups with varying concentrations of AINX, but consistent NfL, something that can also be seen in the DLB group in Fig. 4A

## Discussion

We have shown that AINX is measurable in CSF and is elevated across the spectrum of neurodegenerative diseases. Starting from monoclonal antibody generation, and confirmed reactivity with AINX, we then developed a novel Simoa immunoassay for its quantification.

Overall, validation parameters show a robust and stable assay with variabilities within and between plates well below 15% and an analytical range that allows for detection of the low abundant target analyte. The assay is also flexible enough to allow for sample dilution up to 32-fold and to recover spiked material with almost 100% accuracy. When investigating analyte stability, neither multiple freeze/thaw cycles nor storage at different temperatures (RT and 4 °C) affected calculated concentrations. Furthermore, we tested whether blood contamination would interfere with the accurate measurement of the analyte when, for example, CSF gets contaminated during the lumbar puncture procedure. This proved not to be the case, expanding the potential utility of this assay to acute injury conditions where blood infiltrates the subarachnoid space like subarachnoid hemorrhage or traumatic brain injury, where a CNS-specific neurofilament biomarker might provide insightful information [28, 29].

Being the first immunoassay to accurately detect this analyte in biofluids, there are no reference materials or methods to compare it with. As such, the closest associated protein either in sequence homology or proved functionality is NfL [30]. In this study, NfL was used as reference biomarker for correlations and to assess the potential value of AINX in the context of neurodegeneration biomarkers. This protein recognized as the fourth intermediate filament and being specifically located in the CNS has been, until now, mainly identified by imaging techniques like immunohistochemistry and western blotting, with a small part of prior studies showing its presence in human tissues or fluids [6, 10, 31].

The Gothenburg cohort results show, in a simple comparison between participants with AD and cognitively healthy individuals, the low abundance of AINX in CSF, and the similarities to NfL when it comes to biomarker performance, the latter having been proven multiple times to be a marker for general neurodegeneration [32–34]. In this study, the lower CSF level of AINX compared to NfL, reflects, to some extent, the predicted lower abundance of AINX, as an early intermediate filament, being replaced and complemented by the NFTPs as the brain matures [4]. However, this might also, to some extent, be caused by differences when determining the concentration of the calibrators for each assay.

We hypothesize that, unlike NfL, AINX specifically reflects brain-derived neurodegeneration due to being a CNS-specific protein [19]. Accordingly, it would of course be of interest to compare levels in diseases affecting only the peripheral nervous system (e.g., chronic inflammatory demyelinating polyneuropathy, or Guillain–Barré syndrome) with those affecting peripheral and central nerves (e.g., FTD/amyotrophic lateral sclerosis) as well as research further on conditions such as those examined in this initial study.

Interestingly, when comparing both proteins concentrations, another factor differentiated AINX expression from NfL. There were individual samples, mostly from the AD group, deviating from the correlation line, where NfL was higher independently of AINX. We theorize that AINX might be differentially expressed across the brain, as shown in previous case studies [9, 10]. From the Human Protein Atlas website, we can see that AINX RNA, although expressed in all brain regions, is especially enriched in the parietal lobe, postcentral gyrus [35]. For NfL, a study has shown that it is mostly associated with frontal lobe atrophy [36]. These types of analysis need to be done in future studies, to explore our differential expression theory. For example, immunostaining of brain tissue with well-characterized disease progression with our AINX specific antibodies will help to clarify these findings, as will quantification in larger AD cohorts with a broader spread of disease severities supported by MRI and PET neuroimaging, including defined genetic causes.

The UCL ION cohort gives a general idea of the CSF levels of both proteins across diseases and broadly reaffirmed that AINX and NfL showed similar distribution among diseases, albeit with some key differences. However, the possibility of drawing detailed inter-disease comparisons is limited by the low sample size in each disease. For instance, MS patients showed similar median levels of both AINX and NfL compared to Ctrl. Changes in NfL have been proven to occur depending on disease staging [37], data that is not available for interpretation in our cohort. Also, due to an extremely low number of individuals, bvFTD was excluded from any statistical analysis (*n* = 2). Finally, the DLB group showed a two-cluster pattern for AINX, but not NfL.

There are reports on the performance of NfL to distinguish multiple system atrophy from Lewy Body synucleinopathies [38]. Studies have also shown increased levels of NfL between DLB patients with and without concomitant AD pathology (A+ T+ vs A- T-) [39]. Evaluating AINX in targeted DLB cohorts with defined disease staging and biomarker data might prove useful in respect of differential diagnosis. The correlation between both biomarkers was overall good, without large differences between groups.

This study has some limitations. One of the generated antibodies (Ina1) has a certain degree of cross-reactivity shown in the IP-MS experiments, despite not showing in the ELISA. This might prove impactful in a more sensitive platform like Simoa. However, the assay is still robust, and this issue is solved using the most sensitive antibody as capture in the sandwich layout. The UCL ION cohort has a relatively small number of samples per disease group, which may limit statistical power, though the findings in the groupwise analysis are striking despite the small sample size. Despite this dataset providing a valuable overview of AINX detection and distribution across a range of neurodegenerative conditions, further validation is needed to properly determine clinical application and implementation of AINX. Studying differential diagnosis, case–control discrimination or disease monitoring will be the next steps toward identification of AINX clinical utility together with comparisons with other neurodegeneration biomarkers, data that should be available in future well-powered studies.

This research highlights that AINX is present at low concentration and can be reliably detected in CSF and adult brain tissue using a novel and robust immunoassay. Furthermore, CSF AINX is elevated in several neurodegenerative diseases, indicating its potential as a novel biomarker. Future developments aimed at characterizing AINX in larger and well-defined cohorts, as well as extend the assay reach to blood, will be essential to truly identify the role of this biomarker in the neurodegenerative disease spectrum.

## Supplementary Information

Below is the link to the electronic supplementary material.

Supplementary file1 (PDF 264 KB)

## Data Availability

The datasets used and analyzed during the current study are available from the corresponding author on reasonable request.
