# Supplementary material for: Alpha-internexin as a brain-specific neurodegeneration marker: development and validation of a novel CSF assay
Source: J Neurol. 2025 Oct 6;272(10):676. doi: 10.1007/s00415-025-13428-y (PMC12500819; doi:10.1007/s00415-025-13428-y)
Supplement: Supplementary file 1 — Supplementary file1 (PDF 264 KB) [file 415_2025_13428_MOESM1_ESM.pdf]

Alpha-internexin as a brain-specific neurodegeneration marker: Development and validation of a novel CSF assay.

Francisco J. Meda[1], Anna Dittrich[1,2,3], Ingmar Skoog[1,3], Silke Kern[1,2,3], Claire Leckey[4], Edward J. Wild[5], Ross W. Paterson[4], Gunnar Brinkmalm[1], Ulf Andreasson[1,6], Kaj Blennow[1,6], Henrik Zetterberg[1,4,6,7,8,9,10,11], Hlin Kvartsberg[1,6]

[1] Department of Psychiatry and Neurochemistry, Institute of Neuroscience and Physiology, the Sahlgrenska Academy at the University of Gothenburg, Mölndal, Sweden.
[2] Sahlgrenska Academy, University of Gothenburg, Neuropsychiatric Epidemiology Unit, Gothenburg, Sweden.
[3] Sahlgrenska University Hospital, Department of Neuropsychiatry, Mölndal, Sweden.
[4] UK Dementia Research Institute at UCL, London, United Kingdom.
[5] UCL Huntington's Disease Centre, Institute of Neurology, University College London, United Kingdom.
[6] Clinical Neurochemistry Laboratory, Sahlgrenska University Hospital, Mölndal, Sweden.
[7] Hong Kong Center for Neurodegenerative Diseases, HKCeND, Hong Kong, China.
[8] Wisconsin Alzheimer's Disease Research Center, University of Wisconsin School of Medicine and Public Health, University of Wisconsin-Madison, Madison, WI, USA
[9] Department of Pathology and Laboratory Medicine, University of Wisconsin School of Medicine and Public Health, Madison, WI, United States
[10] Department of Neurodegenerative Disease, Dementia Research Centre, UCL Institute of Neurology, Queen Square, London, United Kingdom.
[11] Centre for Brain Research, Indian Institute of Science, Bangalore, India.

Corresponding author: Francisco Meda, francisco.meda@gu.se

Journal of Neurology

Supplementary data 1

Protein extraction from brain tissue

SDS soluble brain extracts were prepared from brain tissue from the Netherlands Brain Bank.

In brief, brain tissue was removed from -80°C, manually cut, weighted (100 ± 20 mg per extraction) and TBS buffer (20 mM Tris–HCl, 137 mM NaCl, pH = 7.6) equivalent to five times the weight of the tissue added. After this, tissue homogenization was performed using Tissue Lyser II (Qiagen). After centrifugation at 27000 *g* at +4°C for 20 min, the supernatants were collected as the TBS soluble fraction and pellet resuspended in the same volume of 0.5% Triton X-100 with the homogenisation and centrifugation process repeated. Again, supernatant was collected, this time as Triton X-100 soluble, and the pellets resuspended in the same volume of 0.5% SDS buffer followed by homogenisation and centrifugation at +15°C. In the end, supernatants were collected as SDS soluble fraction and stored at -80°C until analysis. Every buffer used in this protocol included cOmplete™, Mini, EDTA-free Protease Inhibitor Cocktail (Roche Diagnostic GmbH) and was cooled to +4°C before usage to reduce protein degradation.

Supplementary data 2

Supplementary data 2. List of tryptic peptides found in IP-MS experiments for both antibodies.

B15		Ina1	
Protein sequence coverage 41%		Protein sequence coverage 85%	
aa-sequence	Peptide	aa-sequence	Peptide
29-39	R.LSGAGGAGGFR.S	29–39	R.LSGAGGAGGFR.S
68-83	R.RPPASDGLDLSQAAAR.T	29–45	R.LSGAGGAGGFRSQSLSR.S
105-111	R.FAVFIEK.V	68–83	R.RPPASDGLDLSQAAAR.T
112-120	K.VHQLETQNR.A	84–91	R.TNEYKIIR.T
121-130	R.ALEAELAALR.Q	92–104	R.TNEKEQLQGLNDR.F
139-145	R.VGELFQR.E	96–104	K.EQLQGLNDR.F
152-161	R.AQLEEASSAR.S	105–111	R.FAVFIEK.V
162-168	R.SQALLER.D	112–120	K.VHQLETQNR.A
169-177	R.DGLAEEVQR.L	121–130	R.ALEAELAALR.Q
202-210	R.DVDGATLAR.L	121–132	R.ALEAELAALRQR.H
217-228	K.VESLLDELAFVR.Q	133–145	R.HAEPSRVGELFQR.E
291-300	K.FANLNEQAAR.S	133–148	R.HAEPSRVGELFQRELR.D
310-316	R.EEIHEYR.R	139–145	R.VGELFQR.E
323-330	R.TIEIEGLR.G	146–161	R.ELRDLRAQLEEASSAR.S
339-346	R.QILELEER.H	149–161	R.DLRAQLEEASSAR.S
378-386	R.EYQDLLNVK.M	152–161	R.AQLEEASSAR.S
399-406	K.LLEGEETR.F	162–168	R.SQALLER.D
431-438	R.ILSATTSK.V	162–177	R.SQALLERDGLAEEVQR.L
439-447	K.VSSTGLSLK.K	162–179	R.SQALLERDGLAEEVQRLR.A
463-482	K.TSQIGESFEEILEETVISTK.K	169–177	R.DGLAEEVQR.L
487-498	K.SNIEETTISSQK.I	188–201	R.GREGAERALKAQQR.D
		202–210	R.DVDGATLAR.L
		202–228	R.DVDGATLARLDLEKKVESLLDELAFVR.Q
		211–228	R.LDLEKKVESLLDELAFVR.Q
		216–228	K.KVESLLDELAFVR.Q
		217–228	K.VESLLDELAFVR.Q
		229–266	R.QVHDEEVAELLATLQASSQAAAEVDVTV AKPDLTSALR.E
		270–278	R.AQYESLAAK.N
		279–288	K.NLQSAEEWYK.S
		289–300	K.SKFANLNEQAAR.S
		291–300	K.FANLNEQAAR.S
		301–317	R.STEAIRASREEIHEYRR.Q
		307–316	R.ASREEIHEYR.R
		307–317	R.ASREEIHEYRR.Q
		310–316	R.EEIHEYR.R
		323–330	R.TIEIEGLR.G
		323–338	R.TIEIEGLRGANESLER.Q
		339–346	R.QILELEER.H

347–366	R.HSAEVAGYQDSIGQLENDLR.N
375–386	R.HLREYQDLLNVK.M
378–386	R.EYQDLLNVK.M
387–397	K.MALDIEIAAYR.K
398–406	R.KLLEGEETR.F
399–406	K.LLEGEETR.F
407–430	R.FSTSGLSISGLNPLPNPSYLLPPR.I
431–447	R.ILSATTSKVSSTGLSLK.K
439–447	K.VSSTGLSLK.K
439–461	K.VSSTGLSLKKEEEEEEASKVASK.K
448–457	K.KEEEEEEASK.V
448–461	K.KEEEEEEASKVASK.K
448–462	K.KEEEEEEASKVASKK.T
462–482	K.KTSQIGESFEEILEETVISTK.K
462–486	K.KTSQIGESFEEILEETVISTKKTEK.S
463–482	K.TSQIGESFEEILEETVISTK.K
463–483	K.TSQIGESFEEILEETVISTKK.T
463–486	K.TSQIGESFEEILEETVISTKKTEK.S
483–499	K.KTEKSNIEETTISSQKI.-
487–498	K.SNIEETTISSQK.I
487–499	K.SNIEETTISSQKI.-

Supplementary data 3

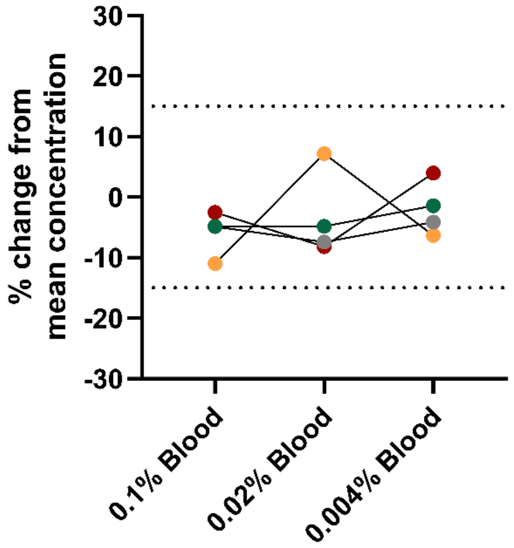

Supplementary data 3. Blood Interference test. Percentage change on concentration of AINX after addition of different volume/volume concentration of whole blood (diluted in 154 mM NaCl). Dotted lines indicate 15% uncertainty limit.

Supplementary data 4

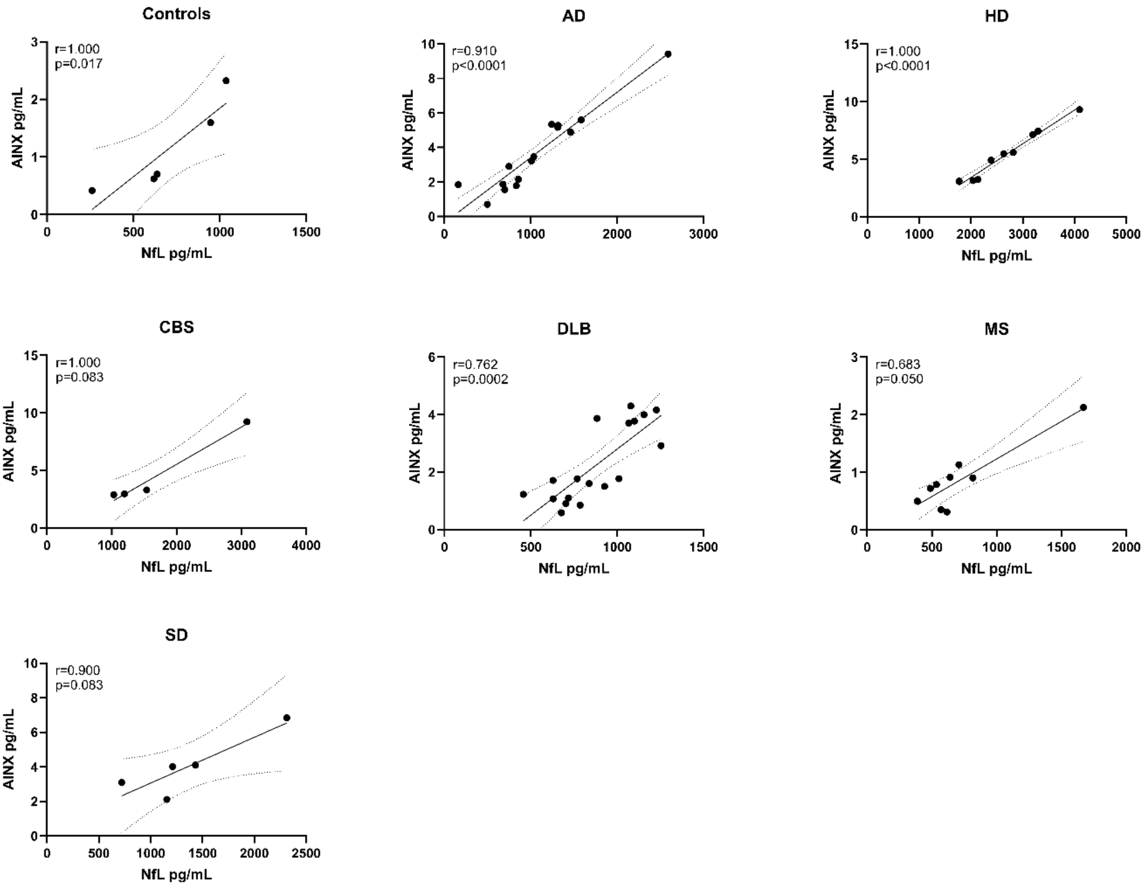

Supplementary data 4. Spearman correlations of every diagnosis group from the UCL ION cohort. Full line represents fitted linear regression and dotted lines indicate 95% confidence intervals. r, correlation coefficient; AD, Alzheimer's Disease (n=15); HD, Huntington's Disease (n=9); CBS, corticobasal syndrome (n=4); SD, semantic dementia (n=5); DLB, dementia with Lewy bodies (n=18); MS, multiple sclerosis (n=9); bvFTD: behavioural variant frontotemporal dementia (n=2); AINX, alpha-internexin; NfL, neurofilament light.
